# Pulmonary Hypertension Secondary to COPD

**DOI:** 10.1155/2012/203952

**Published:** 2012-08-29

**Authors:** Adil Shujaat, Abubakr A. Bajwa, James D. Cury

**Affiliations:** Division of Pulmonary, Critical Care & Sleep Medicine, University of Florida College of Medicine-Jacksonville, 655 West 8th Street, Jacksonville, FL 32209, USA

## Abstract

The development of pulmonary hypertension in COPD adversely affects survival and exercise capacity and is associated with an increased risk of severe acute exacerbations. Unfortunately not all patients with COPD who meet criteria for long term oxygen therapy benefit from it. Even in those who benefit from long term oxygen therapy, such therapy may reverse the elevated pulmonary artery pressure but cannot normalize it. Moreover, the recent discovery of the key roles of endothelial dysfunction and inflammation in the pathogenesis of PH provides the rationale for considering specific pulmonary vasodilators that also possess antiproliferative properties and statins.

## 1. Introduction

Pulmonary hypertension (PH) secondary to chronic obstructive pulmonary disease (COPD) is placed in group 3 of the WHO classification of PH, that is, PH associated with lung diseases and/or hypoxemia (Table 1) [1]. PH in COPD has been variably defined as resting mean pulmonary artery pressure (mPAP) > 20–25 mm Hg. PH in COPD adversely affects survival and exercise capacity and is associated with an increased risk of acute exacerbations. Recent studies have shown that endothelial dysfunction and systemic inflammation also play important roles in the pathogenesis of PH. The recent development of specific pulmonary vasodilators with antiproliferative properties has stimulated an immense interest in studying such drugs in PH secondary to COPD.

## 2. Prevlence of PH in COPD

The prevalence of PH in stable COPD varies from 20 to 91% depending on the definition of PH (mPAP > 20 versus >25 mm Hg), the severity of COPD (forced expiratory volume in the first second: FEV1), and the method of measuring the pulmonary artery pressure (echocardiography versus right heart catheterization) [2–7]. 

In severe COPD patients with or without resting PH, steady-state exercise may raise pulmonary artery pressure (PAP) to about twice the level of its resting value [8]. In severe COPD activities of daily living such as climbing stairs or walking can induce transient PH.

In patients with severe COPD, oxygen saturation may fall during REM sleep by 20–30% [9, 10] and PAP may rise by as much as 20 mm Hg [11].

During an acute exacerbation of COPD, PAP may rise by as much as 20 mm Hg and return to its baseline after recovery [12, 13].

## 3. Significance of PH in COPD

In the era before the widespread availability of long-term oxygen therapy (LTOT) it was well known that the presence of PH was associated with poor prognosis in COPD. However, even on LTOT the best prognostic factor is not the FEV1, nor the degree of hypoxemia or hypercapnia, but the level of mPAP. The 5-year survival rate is only 36% in patients with initial mPAP > 25 mm Hg compared to 62% in those with initial mPAP ≤ 25 mm Hg [14]. Moreover, Weitzenblum et al. [15], who followed up hypoxemic COPD patients with PH on LTOT for a period of 6 years, demonstrated a reversal but not normalization of the PAP. Recently, Zieliński et al. [16] also reported similar findings in a larger study.

PH is also an independent predictor of exercise capacity. Sims et al. [17] found that in 362 severe COPD patients who underwent right-heart catheterization (RHC) as part of evaluation for lung transplantation, higher pulmonary artery pressures were associated with shorter 6-minute walk distance (6MWD) even after controlling for demographics, anthropomorphics, severity of airflow obstruction, and pulmonary artery wedge pressure (PAWP). They found an 11 m decline in 6MWD for every 5 mm rise in mPAP (95% CI 21, 0.7; *P* = 0.04). In another study Cuttica et al. [7] reviewed the records of 1154 COPD patients listed for lung transplantation and found an association between mPAP and 6MWD independent of lung function and PAWP (*β* = −1.33; *P* = 0.01). 

Lastly, it has been shown that a mPAP > 18 mm Hg is associated with an increased risk of severe acute exacerbation in patients with moderate to severe COPD [18].

## 4. Pathophysiology of PH Secondary to COPD

**Figure 1:**
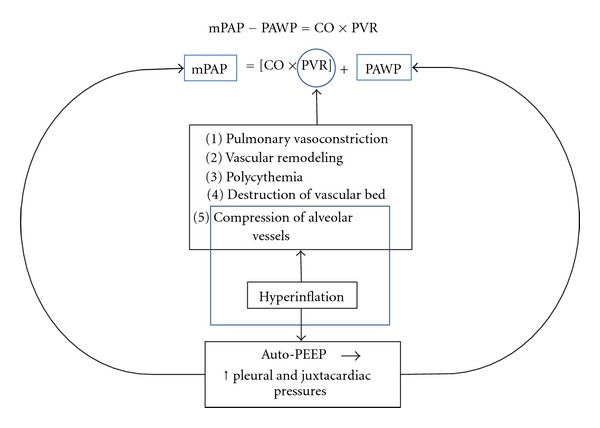
Pathophysiology of PH in COPD. mPAP: mean pulmonary artery pressure, PAWP: pulmonary artery wedge pressure, CO: cardiac output, PVR: pulmonary vascular resistance, PEEP: positive end-expiratory pressure.

In hemodynamic terms PAP depends upon cardiac output (CO), pulmonary vascular resistance (PVR), and pulmonary artery wedge pressure (PAWP) (Figure 1). Resting PH in COPD results predominantly from an elevated PVR whereas PH during exercise results predominantly from an increase in CO in the face of a relatively “fixed” PVR, that is, there is reduced recruitability and distensibility of pulmonary vessels [19]. Hyperinflation increases PVR [20] as well as PAWP [20, 21] and PAP [20], particularly during exercise.

**Figure 2:**
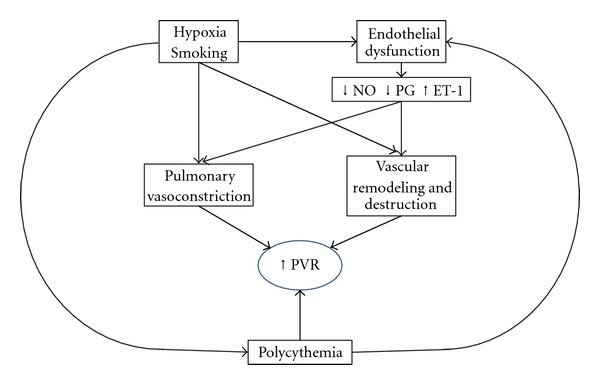
Pathophysiology of elevated PVR in COPD. PVR: pulmonary vascular resistance, NO: nitric oxide, PG: prostaglandin, ET-1: endothelin-1.

Traditionally, elevated PVR in COPD has been considered to be the consequence of hypoxic pulmonary vasoconstriction and vascular remodeling, destruction of the pulmonary vascular bed by emphysema, polycythemia, and hyperinflation. Recently, it has been recognized that endothelial dysfunction and systemic inflammation also play key roles in the pathogenesis of PH (Figure 2). In fact it is believed that the initial event in the natural history of PH in COPD could be endothelial dysfunction caused by cigarette smoke [22].

### 4.1. Pulmonary Vasoconstriction

Hypoxic constriction of the small muscular pulmonary arteries [23] is a protective mechanism to divert blood flow from hypoxic alveoli to better ventilated alveoli and reduce ventilation-perfusion mismatch [24]. However, when alveolar hypoxia is diffuse, such as in severe COPD, it causes generalized pulmonary vasoconstriction and consequently raises the PVR. Persistent hypoxia leads to pulmonary vascular remodeling [25] which contributes to the PVR.

### 4.2. Pulmonary Vascular Remodeling

Vascular remodeling in COPD patients is seen at all stages of the disease and is characterized by intimal fibrosis and proliferation of longitudinal smooth muscle in the muscular pulmonary arteries and arterioles, and neomuscularization of pulmonary arterioles [26–28]. These pulmonary vascular changes also occur in patients with mild COPD and no hypoxia and in smokers with no airway obstruction. This suggests that mechanisms other than hypoxia also play an important role in the pathogenesis of vascular remodeling [29]. 

However, pathologic studies in COPD have not shown complex lesions, which are frequently encountered in patients with pulmonary arterial hypertension [30], such as plexiform lesions (irregular mass of endothelial cells) or angiomatoid lesions, characteristic of severe PH.

### 4.3. Endothelial Dysfunction

The normal endothelium plays an important role in modulating pulmonary vasomotor tone and cellular proliferation. Nitric oxide (NO) produced by endothelial NO synthase (eNOS) has vasodilator and antiproliferative properties. Prostacyclin produced by the activity of prostacyclin synthase is another vasodilator that also protects against vascular remodeling. Countering vasodilatation is endothelium-derived endothelin-1 (ET-1). Endothelial dysfunction caused by smoking, products of inflammation, hypoxia, and shear stress results in altered production of these mediators of tone and/or proliferation, and consequently pulmonary vasoconstriction and vascular remodeling with the latter perpetuating endothelial dysfunction and creating a vicious cycle. In patients with COPD and PH there is a reduction in the synthesis and/or release of NO from the lung [31]. In COPD there is a reduction in the expression of prostacyclin synthase mRNA [32], and in patients with secondary pulmonary hypertension there is an excessive expression of endothelin-1 (ET-1) [33]. Arterial ET-1 also increases shortly after episodes of nocturnal oxygen desaturation in patients with COPD and remains higher during the day in these subjects [34].

### 4.4. Inflammation

Cigarette smoking induces a CD8+ T-lymphocyte infiltration of the adventitia of muscular pulmonary arteries, which correlates with both the endothelium-dependent relaxation and the intimal thickness, suggesting the potential involvement of an inflammatory process in the pathogenesis of pulmonary vascular abnormalities in the early stage of COPD [35].

Systemic inflammation is a known component of COPD [36, 37] and inflammation may contribute to the pathogenesis of PH. Chaouat et al. [38] showed that elevated circulating levels of the proinflammatory cytokine interleukin-6 (IL-6) directly correlated with elevations in mPAP (*r* = 0.39; *P* < 0.001). Moreover, C-reactive protein levels have also been shown to correlate with both PAP and levels of ET-1 [39].

### 4.5. Destruction of the Pulmonary Vascular Bed

Destruction of the pulmonary vascular bed by emphysema reduces the total cross-sectional area of the pulmonary circulation and increases the total PVR when the remaining capacitance vessels are abnormal and unable to accommodate the increased diverted pulmonary blood flow at rest and the increased CO during exercise.

A hypercoagulable state has also been described in patients with COPD [40, 41]. There appears to be an increased frequency of deep venous thrombosis and pulmonary embolism in acute exacerbations of COPD [41–43] and histopathologically thrombotic lesions have been detected in lung tissue from patients with severe emphysema undergoing lung-volume reduction surgery [44]. It is postulated that the inflammatory aspects of the so-called COPD exacerbation may trigger a hypercoagulable state and increase the risk of thrombosis including *in situ* thrombosis.

### 4.6. Polycythemia

Polycythemia not only increases the viscosity of blood and the resistance to blood flow through the pulmonary circulation [45] but also augments hypoxic pulmonary vasoconstriction by causing a local deficiency of NO which may be related to the excessive removal of NO from the pulmonary circulation by the large amount of hemoglobin [46, 47].

### 4.7. Genetic Factors

The pulmonary vascular response to hypoxia is genetically determined. Serotonin (5-hydroxytryptamine, 5-HT) and its transporter (5-HTT) play a role in pulmonary artery smooth muscle cell (PASMC) proliferation and vascular remodeling. The severity of PH in hypoxic COPD patients depends upon 5-HTT gene polymorphism. PH is most severe in patients carrying the LL genotype, which is associated with higher levels of 5-HTT expression in PASMCs [48]. ACE is present in very high concentrations in the lungs and its activity is further increased by hypoxia [49]. ACE is a vasoconstrictor and mediator of PASMC proliferation. The ACE DD genotype is associated with increased circulating and tissue concentrations of ACE. Moreover, the ACE DD genotype is associated with exaggerated PH during exercise in COPD patients [50].

### 4.8. Hyperinflation

Severe emphysema with air-trapping and hyperinflation is associated with intrinsic positive end-expiratory pressure of 5–7.5 cm H_2_O [51]. The positive alveolar pressure throughout respiration contributes to the high PVR [20] as well as increases both PAWP [20, 21] and PAP [20]. This mechanism may assume a more important role in development of PH during exercise and in patients with severe emphysema who are not hypoxemic.

## 5. Right and Left Ventricular Function in PH Secondary to COPD

In response to the increased PVR the right ventricle (RV) gradually undergoes hypertrophy and dilatation-cor pulmonale. This increase in RV end-diastolic volume (RVEDV), that is, preload, to maintain a normal stroke volume (SV) accounts for the reduced RV ejection fraction (EF). RV contractility, as assessed by end systolic pressure-volume relation, is normal in stable COPD patients and the RV operates on an extension of the normal RV function Frank-Starling curve [52]. 

Changes in RV SV must invariably alter left ventricular (LV) preload, because the two ventricles are serially linked through the pulmonary vasculature. LV preload can also be directly altered by changes in RVEDV by the mechanism of ventricular interdependence. The increased RVEDV in cor pulmonale induces a shift of the interventricular septum into the LV and decreases LV diastolic compliance but this does not adversely affect LV SV because the increased RV systolic pressure in cor pulmonale also pushes the septum into the LV towards its free wall to empty the LV. This “help” from the RV in systole tends to preserve LVEF in emphysematous patients with severe RV hypertrophy [53, 54].

More importantly, hyperinflation, particularly during exercise, has the effect of compressing the two ventricles into each other [55, 56]. This decreases RV preload and results in lower SV and CO. Even in less severe COPD the development of hyperinflation during exercise can similarly lead to a reduction in RV preload and CO. 

During an acute exacerbation of COPD, the RV may actually fail, that is, end-diastolic pressure and volume rise and RVEF falls, resulting in peripheral edema and systemic congestion [57, 58]. However, these changes may not be associated with a rise in PAP suggesting that other factors may be operating to reduce RV contractility [57]. Moreover, an acute exacerbation may be associated with peripheral edema in the absence of RV failure [58]. 

The pathogenesis of edema formation in COPD is complex. Renal blood flow is reduced, the renin-angiotensin system is activated, renal dopamine output is reduced, and plasma ANP level is elevated leading to increase in proximal renal tubular sodium reabsorption [59, 60]. Sodium retention is enhanced by hypercapnia and ameliorated by long-term oxygen therapy in hypoxemic patients [61]. True right heart failure is characterized by raised jugular venous pressures, congestive hepatomegaly, and peripheral edema.

## 6. Degree of PH in COPD

Resting PH in stable COPD is usually mild to moderate (mPAP 20–35 mm Hg) and is usually not seen until the disease is advanced (FEV1 < 50%). Severe PH (mPAP > 35–45 mm Hg) is rare (3%–13%) [5–7, 62] and should prompt a search for an additional cause of PH, for example, left heart disease, obstructive sleep apnea (OSA), pulmonary embolism (PE).

### 6.1. Severe “Disproportionate” PH

Recently a group of patients with severe PH (mPAP > 40 mm Hg) and extremely poor prognosis has been recognized. The 5-year survival is 15% versus 55% in those with less severe PH (mPAP 20–40 mm Hg) [62]. Such patients are characterized by mild to moderate airway obstruction, a very low diffusing capacity, severe hypoxemia, and hypocapnia (Table 2) [62]. Thabut et al. [6] have also described a similar group (mPAP > 45 mm Hg). However, such severe PH in COPD in the absence of an alternative explanation is rare (1–3.7%) [6, 62] and suggests the existence of a “vascular phenotype” or concomitant idiopathic pulmonary arterial hypertension.

## 7. Diagnosis of PH in COPD

PH secondary to COPD should be suspected in patients with progressive dyspnea on exertion with stable airway obstruction or in patients with mild to moderate airway obstruction with a very low diffusing capacity, severe hypoxemia, and hypocapnia [6, 62].

A diagnosis of PH in COPD (Table 3) should prompt a search for other causes of PH, particularly left heart dysfunction, OSA, and PE before attributing the PH to COPD.

### 7.1. Clinical Features

The clinical exam lacks sensitivity and specificity. Hyperinflation reduces the yield of cardiac auscultation for the classic signs of PH and right heart failure, that is, loud P2, S3 gallop, the systolic murmur of tricuspid regurgitation. Peripheral edema can be present in the absence of right heart failure in COPD and is not diagnostic of right heart failure. 

### 7.2. CPET

Although cardiopulmonary exercise test (CPET) characteristics show a large overlap in COPD patients with and without PH, the existence of PH in COPD (defined as mPAP > 25 mm Hg) is associated with a significantly reduced ventilatory efficiency during CPET. However, a low SpO_2_ at rest and a further decrease during exercise similarly suggest the presence of PH in COPD [63].

### 7.3. Chest X-Ray

An increase in the diameter of the right descending pulmonary artery to >16 mm on the PA projection, combined with an increase in the diameter of the left descending pulmonary artery of >18 mm on the left lateral projection, has a sensitivity 98% for identifying PH [64].

### 7.4. ECG

Electrocardiographic criteria for the detection of RV hypertrophy have good specificity, but the sensitivity for RV hypertrophy is only 25 to 40%. The criteria include the following: (a) right axis deviation (>100 degrees without right bundle branch block), (b) R or R′ > S in V1, (c) R < S in V6, (d) R in V1 + S in V5 or V6 = 10 mm, (e) R in V1 = 7 mm, (f) R in V1 = 15 mm with right bundle branch block, and (g) right atrial enlargement [52].

However, ECG may reveal other findings such as left atrial enlargement (LAE), left ventricular hypertrophy (LVH), or myocardial infarction in the past that suggests an alternative cause of PH. 

The presence of S1 Q3 T3 (S wave in lead I, Q wave in lead III, and T wave inversion in lead III on ECG—S1, Q3, T3) or right atrial overload pattern (i.e., P wave axis of +90 degrees or more) implies a poor prognosis [65].

### 7.5. ECHO

Hyperinflation precludes optimal visualization of the heart. In a cohort of lung transplant candidates estimation of systolic PAP (sPAP) was possible in only 38% of the 253 patients with COPD. Hyperinflation with a residual volume >150% lessened the likelihood of sPAP estimation. Sensitivity, specificity, negative predictive value (NPV), and positive predictive value (PPV) of sPAP estimated by ECHO for the diagnosis of PH (defined as sPAP >45 mm Hg estimated by ECHO or measured by RHC) were 76, 65, 93, and 32%, respectively. In the absence of sPAP estimation, figures for RV abnormalities were 84, 56, 96, and 22% respectively. It is important to realize that there was a discordance of >10 mm Hg between estimated and measured sPAP in 52% of patients, and in 28%, the discordance was >20 mm Hg [66]. Although the NPV of ECHO is high enough to exclude PH when the heart is adequately visualized, the presence of a high sPAP or RV abnormalities requires confirmation by RHC unless the ECHO shows left heart disease, for example, low LVEF, high LV filling pressure, LVH, left atrial enlargement, valvular incompetence.

Alternatively, transcutaneous Doppler US can be used to measure jugular vein flow velocity. Matsuyama et al. [67] showed that the ratio of diastolic flow (Df) to systemic flow (Sf) velocity showed a significant correlation with mPAP in COPD patients (*r* = 0.844, *P* < 0.0001). The sensitivity was 71.4%, and the specificity 95.3% (cut-off ratio = 1.0). Jugular venous Doppler US could be performed in all patients while other cardiac echo methods could not be performed in all patients. The specificity of the methods used was higher than other cardiac echo methods [67].

### 7.6. BNP

One study of 38 patients with stable COPD, 20 of whom had clinical cor pulmonale, found significant correlation between brain natriuretic peptide (BNP) and ECHO-estimated sPAP (*r* = 0.68, *P* = 0.001) [68]. Elevated BNP also correlated with lower PaO_2_ suggesting that BNP can also be released in response to hypoxia.

### 7.7. Exhaled Nitric Oxide

Clini et al. [69] studied 34 consecutive patients with stable COPD and found that patients with PH (defined as ECHO-estimated sPAP of >35 mm Hg) showed lower values of exhaled nitric oxide compared to those without PH.

### 7.8. Cardiac MRI

This imaging technique produces excellent images of the RV and RV wall thickness shows a high correlation with the mean PAP (*r* = 0.9; *P* < 0.001) [70]. Moreover, it offers many advantages: it is non-invasive, does not involve radiation, and is not affected by hyperinflation. However, it is expensive, not widely available and in some cases claustrophobia can preclude its use.

### 7.9. Chest CT Scan

Enlargement of the main pulmonary artery to ≥29 mm in patients with parenchymal lung disease has been shown to have a sensitivity of 84%, specificity of 75%, PPV of 95%, and positive LR of 3.36 for predicting PH (defined as mPAP > 20 mm Hg) [71].

In another study the ratio of the pulmonary artery to aortic diameter >1 was 70% sensitive and 92% specific for PH (defined as mPAP > 20 mm Hg). The PPV was 96% and the NPV was 52% [72].

Moreover, an increased left atrial area on chest CT could suggest left heart dysfunction as a possible cause of PH [73].

### 7.10. Right Heart Catheterization

RHC remains the “gold standard” for making a diagnosis of PH, accurately determining its severity, and ruling out left heart disease, especially occult LV diastolic dysfunction. An elevated PAWP is not uncommon in severe COPD and does not necessarily imply LV dyfunction [5] as it may be secondary to hyperinflation [21]. Exercise during RHC can help distinguish the cause of an elevated PAWP in COPD. PAWP increases out of proportion to right atrial pressure (RAP) during exercise in comparison to hyperinflation where PAWP and RAP increase proportionately during exercise [74]. Moreover, RHC also measures CO and allows calculation of PVR. Lastly, RHC allows determination of responsiveness to O_2_. However, the invasive nature of the procedure precludes its more widespread use. 

## 8. Natural History of PH in COPD

Kessler et al. [75] studied 131 patients with COPD (mean FEV1 44.6 ± 15.7%) with mild to moderate hypoxemia (PaO_2_ > 60 mm Hg) and without resting PH (mPAP < 20 mm Hg). FEV1 was <35% in 28%, 35–49% in 45%, and ≥50% in 26%. Approximately 25% of the patients developed resting PH during a 6-year followup (mean mPAP 26.8 ± 6.6 mm Hg). More importantly, twice as many patients with exercising PH at the onset developed resting PH over time (32% versus 16%). The average change in mPAP was 0.4 mm Hg per year. Patients with accelerated worsening of resting mPAP differed by a significant worsening of exercising mPAP whereas the changes of FEV1 and PaO_2_ were rather similar. Moreover, patients who developed resting PH had higher resting and exercising mPAP and significantly lower resting and exercising PaO_2_ at baseline [75].

## 9. Treatment of PH Secondary to COPD

The adverse effect of PH on survival and exercise capacity, and the increased risk of severe acute exacerbations caused by PH provide the rationale for treating PH in COPD. The goals of treatment, therefore, are to improve survival, improve exercise tolerance, reduce exacerbations, and improve quality of life. Various approaches to the treatment of PH in COPD are listed in Table 4. Various pulmonary vasodilators used in the treatment of PH in COPD are listed in Table 5.

## 10. Oxygen

LTOT improves survival in stable COPD patients with resting hypoxemia (PaO_2_ < 55 mm Hg) and is associated with a mild improvement in pulmonary hemodynamics [76, 77]. 

In the Medical Research Council trial (*N* = 87), mortality rate at 5 years was 67% in the no-O_2_ group and 45% in the O_2_-treated group (15 h/day). In patients alive at 500 days who received repeat RHC, mPAP increased in the no-O_2_ group (*n* = 21) at an average rate of 2.7 mm Hg/year and remained unchanged in the O_2_-treated group (*n* = 21) [76]. In the Nocturnal Oxygen Therapy (NOT) Trial (*N* = 200), the mortality rate after 1 year was 11.9% in the continuous O_2_ therapy group (averaging 17 h/day) and 20.6% in the nocturnal O_2_ therapy group (averaging 12 h/day). In patients undergoing hemodynamic measurement at baseline and 6 months after enrollment (*n* = 117), mPAP showed a slight rise in the nocturnal O_2_ therapy group and a slight fall (at an average of 3 mm Hg/year) in the continuous O_2_ therapy group. PVR decreased by 11.1% in the continuous O_2_ therapy group but increased by 6.5% in the nocturnal O_2_ therapy group [78].

Unfortunately not all patients with COPD who meet criteria for LTOT benefit from it. Ashutosh et al. [79] showed that patients who exhibited a significant drop in mean PAP of ≥5 mm Hg after acute O_2_ therapy (28% for 24 h) had an 88% 2-year survival compared to 22% in nonresponders when both groups of patients were subsequently treated with continuous LTOT [79]. Of note, room air VO_2_ max provided the same information in that study with 6.5 mL/kg/min being the cut-off that distinguished responders from nonresponders [79].

Similarly, even in the landmark NOT trial O_2_ therapy resulted in an improved survival only in patients whose baseline SVI was >30 mL/beat/m^2^ (in the continuous O_2_ group) or PVR was <400 dyne·s·cm^−5^ (in the nocturnal O_2_ group) [78].

Weitzenblum et al. who followed up 16 hypoxemic COPD patients on LTOT for a period of 6 years demonstrated a reversal but not normalization of the PAP [15].

Moreover, supplemental O_2_ during exercise decreases PAP and increases exercise tolerance even in COPD patients with mild resting hypoxemia (PaO_2_ > 60 mm Hg) and moderate-to-severe airflow obstruction [80]. This effect was found to be the result of inhibition of hypoxic pulmonary vasoconstriction and reduction in air trapping (indicated by the difference in slow and forced vital capacity). Others have also shown that supplemental O_2_ reduces dynamic hyperinflation and consequently the PAP and PAWP [20, 81]. Supplemental O_2_ during exercise also improves RV function [82].

Lastly, O_2_ therapy abolishes the nocturnal rise in PAP acutely [83] as well as decreases PAP in the long-term in COPD patients with PH and daytime PaO_2_ > 60 mm Hg who experience nocturnal desaturation [84].

## 11. Nonspecific Pulmonary Vasodilators

Various vasodilators: calcium channel blockers,  *β*2-agonists, nitrates, angiotensin converting enzyme inhibitors, and  *α*1-antagonists were studied in the 80s. Most of them caused a modest decrease in mPAP accompanied by an increase in CO and a decrease in PVR but they were also associated with systemic hypotension and worsening of ventilation-perfusion mismatch that in some cases was not offset by the increase in CO [85].

## 12. Specific Pulmonary Vasodilators

The recent discovery of endothelial dysfunction resulting in the altered production of mediators of tone and/or proliferation, and consequently pulmonary vasoconstriction and vascular remodeling, provides the rationale for considering specific pulmonary vasodilators that also possess antiproliferative properties. 

### 12.1. Inhaled Nitric Oxide

Inhaled nitric oxide (iNO) is a more potent vasodilator than O_2_. However, when used alone iNO worsens ventilation-perfusion imbalance. In a randomized controlled trial (RCT) 40 patients with severe COPD (mean FEV1 1.19 ± 0.6 L) and PH (mPAP > 25 mm Hg) who were receiving LTOT were randomized to pulsed iNO (delivered via a novel device NOXXI; Messer, Austria) plus O_2_ or O_2_ alone for 3 months [86]. There was a significant improvement in mPAP, PVR, and CO. The mPAP decreased from 27.6 to 20.6 mm Hg (*P* < 0.001); PVR decreased from 276.9 to 173 dyne·s·cm^−5^ (*P* < 0.001). Systemic hemodynamics and left heart function remained unchanged. PaCO_2_ decreased significantly in the treatment group, suggesting improved perfusion of the better ventilated areas. Significant methemoglobinemia was not seen. Although this study shows a promising role for iNO in stable COPD patients with PH, it is important to realize that iNO needs to be delivered in a pulsed manner to limit the formation of nitrogen dioxide and to avoid worsening ventilation-perfusion mismatch, and such delivery requires a more practical device. 

### 12.2. Inhaled Iloprost

In a study by Dernaika et al. [87] 10 males with FEV1 < 65% with Pa O_2_ 60–75 mm Hg and PH (defined as sPAP > 35 mm Hg plus RV dilatation and/or RV hypertrophy on ECHO) were evaluated before and after inhaling 2 doses of iloprost (2.5 *μ*g). PFT, ABG, 6MWT and ventilatory equivalents for O_2_ (VE/VO_2_) and CO_2_ (VE/VCO_2_) were performed at baseline, 30 min following each dose of iloprost, and 2 h after the second dose. Iloprost was associated with improved ventilation-perfusion matching and exercise tolerance. The 6MWD increased by 49.8 ± 35 m (*P* = 0.02).

### 12.3. Lessons Learnt from the Trials of Inhaled Pulmonary Vasodilators

In patients with severe COPD and resting mPAP > 25 mm Hg inhaled NO and O_2_ improve pulmonary hemodynamics and ventilation-perfusion mismatch. The recent development of a lightweight (approximately 4 kg) and portable pulsed delivery system INOpulse DS, that also eliminates the need for calibration or monitoring of NO or NO_2_, offers the possibility of using inhaled NO in COPD patients with PH. However, this promising device has not been studied in COPD or PH.

In patients with COPD with FEV1 < 65% and ECHO-estimated resting sPAP > 35 mm Hg iloprost alone improves ventilation-perfusion mismatch and 6MWD. However, its effects last only 2 hours. Another inhaled prostaglandin treprostinil, which is now available and has a longer duration of action, may be a more feasible option. However, it has not been studied in COPD.

### 12.4. Sildenafil

Alp et al. [88] were the first to report on the acute and long-term effects of sildenafil in COPD. They showed that in 6 patients with COPD with FEV1 < 50% and PH (mPAP 29.5 ± 5.2 mm Hg) sildenafil 50 mg IV, once followed by 50 mg PO BID for 3 months, resulted in significant improvement in both hemodynamics and 6MWD. The mean 6MWD increased by 82 m (from 351 ± 49 to 433 ± 52 m) after 3 months.

However, Holverda et al. [89] failed to show similar results in two studies. They studied the acute effects of a single oral dose of sildenafil 50 mg in 18 patients with GOLD stage II–IV and showed that regardless of the mPAP at rest, sildenafil attenuated the increase in mPAP during submaximal exercise but this was neither accompanied by enhanced SV and CO, nor by improved exercise capacity. However, only 11 patients had PH: 5 at rest (mPAP > 25 mm Hg) and 6 with PH on exercise (mPAP > 30 mm Hg).

The same group went on to study the effects of sildenafil 50 mg PO TID for 3 months in 15 patients with GOLD stage II–IV and reported similar results—neither SV nor exercise capacity improved [90]. However, again, not all patients had PH—only 9 had PH: 5 at rest (mPAP > 25 mm Hg) and 4 on exercise (mPAP > 30 mm Hg).

In a randomized dose comparison trial of sildenafil 20 versus 40 mg in 20 patients with COPD and resting PH (mPAP > 20 mm Hg) both doses improved pulmonary hemodynamics at rest and during exercise but this was accompanied by worsening hypoxemia albeit only at rest [91]. Interestingly, there was also noted to be a slight but statistically significant improvement in FEV1 and forced vital capacity (FVC). Although such a bronchodilatory effect of sildenafil has also been reported in two patients in the literature [92] and is probably mediated through its inhibition of the phosphodiesterase-5 enzyme [93], it has not been evaluated in a controlled manner.

On the other hand, in a double blind RCT of 33 patients with severe COPD and ECHO-estimated sPAP > 40 mm Hg Rao et al. [94] showed that the median 6MWD improved by 191 m and sPAP by 12 mm Hg after sildenafil 20 mg PO TID for 3 months (*P* < 0.05). 

### 12.5. Bosentan

In a double blind RCT of 30 patients with severe to very severe COPD Stolz et al. [95] showed that bosentan 125 mg PO BID for 3 months not only failed to improve exercise capacity but also deteriorated hypoxemia and functional status. It is important to keep in mind that only 14 of 20 patients in the bosentan group and 6 of 10 patients in the placebo group had PH at rest (defined as ECHO-estimated sPAP > 30 mm Hg without adding central venous pressure).

On the contrary, in another RCT, this one of 40 patients with COPD and PH (mPAP > 25 mm Hg and PAWP < 15 mm Hg), Valerio et al. [96] showed that bosentan 125 mg PO BID for 18 months resulted in a significant improvement in hemodynamics, 6MWD and BODE index: mPAP from 37± to 31 ± 6 mm Hg, PVR from 442 ± 192 to 392 ± 180 dyne·s·cm^−5^, 6MWD from 256 ± 118 to 321 ± 122 m, and BODE index from 6.6 ± 2.8 to 5.5 ± 3 units. Most patients in stage IV, who made up 30% of the study population and were characterized by high BODE index, WHO functional class IV, no reversibility with O2, and higher increases in PAP and PVR during exercise, did not improve but in all such patients the treatment stopped the progressive worsening of hemodynamics.

### 12.6. Lessons Learnt from the Trials of Oral Specific Pulmonary Vasodilators (Tables 6 and 7)

**Figure 3:**
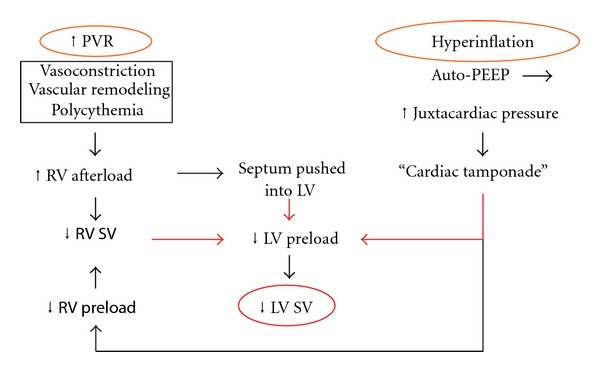
Pathophysiology of reduced SV in COPD. SV: stroke volume, PVR: pulmonary vascular resistance, RV: right ventricular, LV: left ventricular, PEEP: positive end-expiratory pressure.

In patients with severe COPD *and* resting mPAP < 25 mm Hg pulmonary vasodilator therapy may improve PAP during exercise but does not improve SV and CO or exercise capacity. This is probably because hyperinflation plays a predominant role in the pathophysiology of reduced SV and CO in such patients (Figure 3). Severe hyperinflation with inspiratory capacity to total lung capacity (IC/TLC) ratio <25% causes a “tamponade” effect on the heart and reduces RV preload [55, 56, 97] whereas any reduction in RV afterload that may result from pulmonary vasodilatation is of no avail and the SV is limited particularly during exercise. Therefore, pulmonary vasodilators should be neither studied nor used in COPD patients with mild resting PH (mPAP < 25 mm Hg or ECHO-estimated sPAP < 40 mm Hg) or in COPD patients with PH only on exercise.

On the other hand, in patients with COPD and resting mPAP > 25 mm Hg or ECHO-estimated sPAP > 50 mm Hg pulmonary vasodilator therapy improves pulmonary hemodynamics and 6MWD. However, more research is needed to recommend the use of pulmonary vasodilators in PH secondary to COPD. Although COPD patients with severe PH (mPAP > 35–45 mm Hg) who probably represent a “vascular phenotype” or have concomitant IPAH will benefit the most from pulmonary vasodilator therapy, it may be worthwhile to try such therapy in COPD patients with less severe PH (mPAP 25–35 mm Hg) especially if hyperinflation is not playing a significant role. Although it has not been evaluated, pulse oximeter plethysmography waveform analysis to identify “pulsus paradoxus” may be a simpler way of identifying patients with severe hyperinflation with IC/TLC ratio <25%. 

It is important to keep in mind that even specific pulmonary vasodilators can worsen ventilation-perfusion mismatch and hypoxemia at rest that may or may not be offset by an increase in CO. Lastly, lack of acute responsiveness to pulmonary vasodilators indicates a more altered vasculature that may respond to a longer course of therapy or to statins.

## 13. Statins

Statins have anti-inflammatory, antioxidant, and antithrombogenic effects and restore endothelial function [98]. Moreover, statins can reduce the synthesis of ET-1 at the transcriptional level [99].

In a double-blind parallel design study [100], 53 patients with COPD and ECHO-estimated sPAP > 35 mm Hg were randomly assigned to receive either pravastatin 40 mg daily or placebo for 6 months. Exercise time increased significantly 52% from 660 ± 352 to 1006 ± 316 seconds (*P* < 0.0001) in the treatment group. ECHO-estimated sPAP decreased significantly from 47 ± 8 to 40 ± 6 mm Hg. There was also significant improvement in Borg dyspnea score. 

In an animal study, Wright et al. [101] studied the effects of simvastatin in guinea pigs exposed to cigarette smoke for 6 months. In half of the animals simvastatin was introduced after 3 months. Cigarette smoke increased the sPAP after approximately 4 weeks. Simvastatin returned the pressure to control levels within 4 weeks of starting treatment, and ameliorated smoke-induced small arterial remodeling as well as emphysema measured both physiologically and morphometrically at 6 months, but did not prevent smoke-induced small airway remodeling either physiologically or morphologically. In precision-cut lung slices simvastatin reversed small arterial endothelial dysfunction and partially reversed smoke-induced loss of vascular NO generation.

Both studies show prospects for the use of statins in COPD and warrant more research.

## 14. Diuretics

Diuretics reduce right ventricular dilatation and improve its contractility and also reduce extravascular lung water [102]. They should be used cautiously as they can cause intravascular volume depletion that may deprive the RV of adequate preload to maintain a normal SV. 

## 15. Phlebotomy

Phlebotomy is usually indicated in patients with polycythemia not responding to LTOT. In a small study of 7 patients with stable severe COPD (FEV1 33 ± 3% of predicted) and PH, Borst et al. [103] showed that isovolemic phlebotomy resulted in improvement in pulmonary hemodynamics, gas exchange, and exercise tolerance. The patients were phlebotomized 5-6 times over a period of 3 months with substitution of 6% hydroxyethyl starch (molecular weight 40,000). This resulted in a stepwise reduction of the hematocrit from 53.3 ± 2.6 to 45.8 ± 3.1%. Mean PAP decreased from 30 ± 3 to 22 ± 2 mm Hg and PaO_2_ increased from 63.2 ± 2.2 to 71.8 ± 3.7 mm Hg at rest. During peak exercise, mPAP decreased from 59 ± 7 to 53 ± 7 mm Hg and PaO_2_ increased from 54.0 ± 5.7 to 63.2 ± 2.4 mm Hg after hemodilution. Peak oxygen consumption rose from 573 ± 84 to 750 ± 59 mL/min, corresponding to an increase in CI from 4.25 ± 0.5 to 5.88 ± 0.76 liters/min/m^2^. PVR fell from 345 ± 53 to 194 ± 32 dyne·s·cm^−5^. The patients' peak exercise capacity increased from 9.2 ± 2.0 before to 13.5 ± 3.2 kJ at the end of the study (*P* < 0.05 for all differences).

## 16. “Bloodless Phlebotomy”

Activation of the renin-angiotensin system may contribute to polycythemia in COPD [104]. Plasma renin and aldosterone levels are increased in such patients when matched with controls for hypoxemia. The mechanism of action is serum-erythropoietin-independent. In a small study, the angiotensin receptor blocker (ARB) losartan was used in weekly escalating doses to a maximum of 100 mg daily for 4 weeks in 9 stable severe COPD patients with polycythemia (hematocrit >52%). The regimen caused a significant reduction in the hematocrit of all patients from 56 ± 0.9% to 46 ± 0.7% (*P* < 0.001). The higher the baseline value, the greater the reduction in hematocrit (*r* = 0.7085; *P* < 0.05) [105]. At 3 months after discontinuation of losartan the hematocrit increased to 50 ± 0.7%. Similarly, in an RCT of 60 patients with severe COPD another ARB irbesartan also induced a significant reduction in hematocrit [106]. Of note, however, neither study evaluated pulmonary hemodynamics, gas exchange or exercise tolerance which makes it difficult to draw any meaningful conclusions. 

Although it is tempting to speculate that such a “bloodless” phlebotomy may also result in improvement in pulmonary hemodynamics, gas exchange, and exercise tolerance, it is important to realize that ARBs are also non-specific vasodilators that can cause a modest decrease in mPAP as well as worsen PaO_2_. In fact in a double-blind RCT of COPD patients with transtricuspid pressure gradient (TTPG) >30 mm Hg more patients in the losartan group (50%) than in the placebo group (22%) showed a clinically meaningful reduction in TTPG at any time point during the 12-month period, and these effects seemed more marked in patients with higher baseline TTPG. There were no clear improvements in exercise capacity or symptoms, though [107].

## 17. Lung Volume Reduction Surgery (LVRS)

Although lung volume reduction surgery (LVRS) is contra-indicated in COPD patients with severe PH (mPAP > 35 mm Hg), the reduction in hyperinflation and improvement in gas exchange resulting from such surgery are expected to result in an improvement in PAP in patients with less severe PH. On the other hand excision of some viable pulmonary vascular bed may have the adverse effect of worsening PVR. In fact the few studies of pulmonary hemodynamics before and after LVRS have shown that the mPAP remains unchanged after such surgery [108–111]. Earlier and smaller studies showed that mPAP remains unchanged because CO improves when PVR falls after LVRS [108–110]. In contrast, the most recent and largest study of pulmonary hemodynamics before and after LVRS, which was a cardiac substudy of the national emphysema treatment (NET) trial, did not show any significant change in CO [111]. Reasons for the discrepancy between the results of the earlier studies and the NET trial are not clear, but could be due to differences in patient selection, or surgical methods. Moreover, unlike the other studies, the NET trial did not evaluate pulmonary hemodynamics during exercise.

## 18. Lung Transplantation 

PH secondary to COPD is an indication for lung transplantation. Bjortuft et al. [112] investigated a group of 24 patients, including 19 with COPD, who underwent single lung transplantation. The majority (15 out of 24) of patients had mild-to-moderate PH at the onset and in these patients mPAP significantly decreased from 28 ± 1 to 18 ± 1 mm Hg after transplantation; there was a similar decrease in PVR. These results were maintained after 2 yrs of followup. Therefore, COPD patients with PH normalize pulmonary haemodynamics after single lung transplantation.

## 19. Conclusion

The pathophysiology of PH in COPD is complex. A diagnosis of PH in COPD should prompt a search for other causes of PH, particularly left heart dysfunction, OSA, and PE before attributing the PH to COPD. PH in COPD adversely affects survival and exercise capacity and is associated with an increased risk of severe acute exacerbations. Unfortunately not all patients with COPD who meet criteria for LTOT benefit from it. Even in those who benefit from LTOT, such therapy may reverse PAP but cannot normalize it. Moreover, the recent discovery of the key roles of endothelial dysfunction and inflammation in the pathogenesis of PH provides the rationale for considering specific pulmonary vasodilators that also possess antiproliferative properties and statins. Studies of pulmonary vasodilators and statins for PH secondary to COPD appear to show a promising role for such therapy in patients with more than mild PH (mPAP > 25 mm Hg) and warrant more research. Success of pulmonary vasodilator therapy appears to depend upon the degree of PH and the severity of hyperinflation. Such therapy is more likely to be successful when PH is moderate to severe (mPAP > 25 mm Hg) and hyperinflation is not playing a significant role, that is, IC/TLC is >25%. Although stable COPD patients with severe PH (mPAP > 35–45 mm Hg) who probably represent a “vascular phenotype” or have concomitant IPAH warrant pulmonary vasodilator therapy, it may be worthwhile to try such therapy in stable COPD patients with less severe PH (mPAP 25–35 mm Hg) especially if hyperinflation is not severe. On the other hand such therapy should be avoided when PH is mild or only during exercise or hyperinflation is playing a significant role, that is, IC/TLC is <25%. Future studies of pharmacotherapy should focus on patients with PH with mPAP > 25 mm Hg and IC/TLC > 25%.

## Figures and Tables

**Table 1:** Updated clinical classification of pulmonary hypertension (Dana Point, 2008) [1].

(1) Pulmonary arterial hypertension (PAH) (1.1) Idiopathic PAH (1.2) Heritable (1.2.1) BMPR2 (1.2.2) ALK1, endoglin (with or without hereditary hemorrhagic telangiectasia) (1.2.3) Unknown (1.3) Drug- and toxin-induced (1.4) Associated with (1.4.1) Connective tissue diseases (1.4.2) HIV infection (1.4.3) Portal hypertension (1.4.4) Congenital heart disease (1.4.5) Schistosomiasis (1.4.6) Chronic hemolytic anemia (1.5) Persistent pulmonary hypertension of the newborn	

(i) Pulmonary venoocclusive disease (PVOD) and/or pulmonary capillary hemangiomatosis (PCH)	

(2) Pulmonary hypertension owing to left-heart disease (2.1) Systolic dysfunction (2.2) Diastolic dysfunction (2.3) Valvular disease	

(3) Pulmonary hypertension owing to lung disease and/or hypoxia (3.1) Chronic obstructive pulmonary disease (COPD) (3.2) Interstitial lung disease (3.3) Other pulmonary diseases with mixed restrictive and obstructive pattern (3.4) Sleep-disordered breathing (3.5) Alveolar hypoventilation disorders (3.6) Chronic exposure to high altitude (3.7) Developmental abnormalities	

(4) Chronic thromboembolic pulmonary hypertension (CTEPH)	

(5) Pulmonary hypertension with unclear multifactorial mechanisms (5.1) Hematologic disorders: myeloproliferative disorders, splenectomy (5.2) Systemic disorders: sarcoidosis, pulmonary Langerhans cell histiocytosis, lymphangioleiomyomatosis, neurofibromatosis, vasculitis (5.3) Metabolic disorders: glycogen storage disease, Gaucher disease, thyroid disorders (5.4) Others: tumoral obstruction, fibrosing mediastinitis, chronic renal failure on dialysis	

Simonneau [1].

**Table 2:** Comparison of 2 groups of COPD patients with PH.

	Severe PH Group (mPAP ≥ 40 mm Hg) *N* = 11	Less severe PH (mPAP 20–40 mm Hg) *N* = 16	*P* value
FEV1 (% predicted)	50 (44–56)	27 (23–34)	<0.01
DLCO (mL/min/mm Hg)	4.6 (4.2–6.7)	10.3 (8.9–12.8)	<0.01
PaO_2_ (mm Hg)	46 (41–53)	56 (54–64)	<0.01
PaCO_2_ (mm Hg)	32 (28–37)	47 (44–49)	<0.01
RAP (mm Hg)	7 (5–9)	3 (1.3–4)	<0.01
mPAP (mm Hg)	48 (46–50)	25 (22–27)	<0.01
PAWP (mm Hg)	6 (4–7)	7 (6.5-7.5)	NS
CI (L/min/m^2^)	2.3 (1.8–2.5)	2.8 (2.4–3.1)	<0.01
TPR (Wood units/m^2^)	21.3 (17.6–26.6)	9 (7.4–9.9)	<0.01

Table adapted from [63].

PH: pulmonary hypertension, FEV1: forced expiratory volume in the first second, DLCO: diffusing capacity for carbon monoxide, PaO_2_: arterial oxygen tension, PaCO_2_: arterial carbon dioxide tension, RAP: right atrial pressure, mPAP: mean pulmonary artery pressure, CI: cardiac index, TPR: total pulmonary resistance.

**Table 3:** Various approaches to the diagnosis of PH in COPD.

Modality	Advantages	Disadvantages
ECG	Noninvasive, cheap, and readily available. High specificity for RVH. ECG may reveal other findings such LAE, LVH, or old MI that suggests an alternative cause of PH	Absence of RVH does not rule out PH.

CXR	Non-invasive, cheap, and readily available. An ↑ in the diameter of the right descending pulmonary artery to >16 mm on the PA projection, combined with an ↑ in the diameter of the left descending pulmonary artery of >18 mm on the left lateral projection, has a high sensitivity of 98% for PH	Normal-sized pulmonary artery does not rule out PH.

BNP	Requires only a blood draw, is cheap and readily available.	↑ BNP also correlated with lower PaO_2_ suggesting that BNP can also be released in response to hypoxia. More studies are needed.

eNO	Non-invasive.	Expensive, not widely available and has been tested in only one study.

ECHO	High NPV of sPAP or RV abnormalities (93% and 96%, resp.) makes it an excellent screening test. Moreover, it provides additional data for example, LVEF, LV filling pressures, valvular function.	Hyperinflation may preclude optimal visualization of the heart. Although the NPV is high enough to exclude PH, the presence of a high sPAP or RV abnormalities requires confirmation by RHC.

Chest CT	Non-invasive, widely available. High PPV of 95%-96% for PH. LAE could suggest left heart dysfunction.	Expensive. Radiation exposure. Normal sized pulmonary artery does not rule out PH.

Cardiac MRI	Non-invasive, does not involve ionizing radiation, and is not affected by hyperinflation.	Expensive, not widely available and in some cases claustrophobia can be a problem.

RHC	“Gold standard” Confirms diagnosis. Determines severity. Distinguishes occult LV dysfunction from hyperinflation when PAWP is ↑. Measures CO and allows calculation of PVR. Determines responsiveness to O_2_.	Invasive. Interpretation of pressures may be difficult when there are large respiratory swings.

PH: pulmonary hypertension, EKG: electrocardiography, RVH: right ventricular hypertrophy, LAE: left atrial enlargement, LVH: left ventricular hypertrophy, MI: myocardial infarction, CXR: chest X-ray, BNP: brain natriuretic peptide, PaO_2_: arterial oxygen tension, eNO: exhaled nitric oxide, NPV: negative predictive value, ECHO: echocardiography, sPAP: systolic pulmonary artery pressure, RV: right ventricular, LVEF: left ventricular ejection fraction, LV: left ventricular, RHC: right heart catheterization, CT: computerized axial tomography, PPV: positive predictive value, MRI: magnetic resonance imaging, CO: cardiac output, PVR: pulmonary vascular resistance, PAWP: pulmonary artery wedge pressure, RAP: right atrial pressure, O_2_: oxygen.

**Table 4:** Various approaches to the treatment of PH in COPD.

Counteract hyperinflation	Counteract pulmonary vasoconstriction	Counteract vascular remodeling	Counteract polycythemia
Bronchodilators	O_2_	O_2_	O_2_
O_2_	Pulmonary vasodilators	Pulmonary vasodilators	Phlebotomy
Sildenafil		Statins	ARB
LVRS (unless PH severe)			
Lung transplantation			
Smoking cessation			

PH: pulmonary hypertension, O_2_: oxygen, LVRS: lung volume reduction surgery, ARB: angiotensin receptor blocker.

**Table 5:** Various pulmonary vasodilators studied for the treatment of PH in COPD.

Inhaled	Systemically delivered	
	Nonspecific	Specific
O_2_	CCB: nifedipine, felodipine	PDE5 I: sildenafil
NO	*α*-1 antagonist: prazosin	ETRA: bosentan
PG: iloprost	ACEI: captopril	

PH: pulmonary hypertension, O_2_: oxygen, NO: nitric oxide, PG: prostaglandin, CCB: calcium channel blocker, ACEI: angiotensin converting enzyme inhibitor, PDE5 I: phosphodiesterase 5 inhibitor, ETRA: endothelin receptor antagonist.

**Table 6:** Summary of the effects of pulmonary vasodilators in the published studies of such drugs in COPD patients.

First author	Alp et al. [88]	Holverda et al. [89]	Rietema et al. [90]	Stolz et al. [95]	Valerio et al. [96]	Blanco et al. [91]	Rao et al. [94]
Year of publication	2006	2008	2008	2008	2009	2010	2011
Country	Germany	Netherlands	Netherlands	Switzerland	Italy	Spain	India
Drug	Sildenafil	Sildenafil	Sildenafil	Bosentan	Bosentan	Sildenafil	Sildenafil
Dose	50 mg BID	50 mg	50 mg TID	125 mg BID	125 mg BID	20 mg vs 40 mg	20 mg TID
Duration	3 months	Acute effects	3 months	3 months	18 months	Acute effects	3 months
Total *N *	5	18	15	20	20	20	17
*N* with PH	5	11	9	14	20	12	17
*N* with resting PH	5	5	5	14	20	12	17
PAP	↓	↓ r + e		No Δ	↓	↓ r + e	↓
CO	NA	No Δ r + e	No Δ r + e	No Δ	No Δ	↑ r + e	
PVR	↓	No Δ r + e		No Δ	↓	↓ r + e	
SpO_2_PaO_2_		↓ r + e	No Δ r + e	↓ r, No Δ e	No Δ	↓ r, No Δ e	
6MWD (m) at baseline	351 ± 49		385 ± 135	339 ± 81	257 ± 150	396 ± 114	269 ± 140
6MWD (m) after treatment	↑ to 433 ± 52		↑ to 396 ± 116	↑ to 329 ± 94	↑ to 321 ± 122	NA	↑ by 191 ± 127

mg: milligrams, BID: twice a day, TID: three times a day, vs: versus, *N*: total number of patients who received the study drug, *N* with PH: number of patients with pulmonary hypertension, PH: pulmonary hypertension, PAP: pulmonary artery pressure, CO: cardiac output, PVR: pulmonary vascular resistance, SpO_2_: oxygen saturation by pulse oximetry, PaO_2_: arterial oxygen tension, 6MWD: six-minute walk distance, m: meters, NA: not available or not applicable, r + e: rest and exercise, Δ: change, r: rest, e: exercise.

**Table 7:** Baseline characteristics of the patients who received pulmonary vasodilators in the published studies of such drugs in COPD.

First author	Alp et al. [88]	Holverda et al. [89]	Rietema et al. [90]	Stolz et al. [95]	Valerio et al. [96]	Blanco et al. [91]	Rao et al. [94]
Year of publication	2006	2008	2008	2008	2009	2010	2011
Drug	Sildenafil	Sildenafil	Sildenafil	Bosentan	Bosentan	Sildenafil	Sildenafil
Dose	50 mg BID	50 mg	50 mg TID	125 mg BID	125 mg BID	20 mg vs 40 mg	20 mg TID
Duration	3 months	Acute effects	3 months	3 months	18 months	Acute effects	3 months
Total *N *	5	18	15	20	20	20	17
*N* with PH	5	11	9	14^†^	20	12	17
*N* with resting PH*	5	5	5	14^†^	20	12	17
Age (years)	45–64	66 ± 9	65 ± 2	69.5 ± 8.8	66 ± 9	64 ± 7	60 ± 7
sPAP (mm Hg)				32^‡^			53 ± 12
**mPAP (mm Hg)**	**29.5 ± 5.2**	23 ± 10	22 ± 9		**37 ± 5**	**27 ± 10**	***33.8 ± 9.2*** ^§^
CO (L/min)		5.5 ± 1.0	5.4 ± 1.7	2.45 ± 0.4	2.8 ± 0.7	4.9 ± 0.95	
CI (L/min/m^2^)						2.7 ± 0.44	
PVR (dynes.s.cm^−5^)	373 ± 118	280 ± 180	259 ± 166	158 ± 30	442 ± 192	339 ± 165	
**FEV1 (% predicted)**	16–48	52 ± 26	49 ± 24	38 ± 13	37 ± 18	35 ± 11	32.5 ± 11
TLC (% predicted)		126 ± 15	125 ± 16	126 ± 15	132 ± 6	114 ± 19	
DLCO (% predicted)		46 ± 17	48 ± 16	37 ± 18	34 ± 7	44 ± 17	
SpO_2_ (%)		93 ± 4	95 ± 2	93 ± 3			
PaO_2_ (mm Hg)			74 ± 13		57 ± 10	64 ± 11	
PaCO_2_ (mm Hg)			39 ± 6		46 ± 8	38.4 ± 4.5	
6MWD (meters)	351 ± 49		385 ± 135	339 ± 81	257 ± 150	396 ± 114	269 ± 140

mg: milligrams, BID: twice a day, TID: three times a day, vs: versus, *N*: total number of patients who received the study drug, *N* with PH: number of patients with pulmonary hypertension, PH: pulmonary hypertension, sPAP: systolic pulmonary artery pressure estimated by echocardiography, mPAP: mean pulmonary artery pressure measured by right heart catheterization, CO: cardiac output, CI: cardiac index, PVR: pulmonary vascular resistance, FEV1: forced expiratory volume in the first second, TLC: total lung capacity, DLCO: diffusing capacity for carbon monoxide, SpO_2_: oxygen saturation by pulse oximetry, PaO_2_: arterial oxygen tension, PaCO_2_: arterial carbon dioxide tension, 6MWD: six-minute walk distance.

*resting PH defined as mPAP > 25 mm Hg or ECHO estimated sPAP > 40 mm Hg unless specified otherwise—see below:

^†^PH was defined as estimated sPAP > 30 mm Hg without adding central venous pressure (CVP).

^‡^Estimated sPAP without adding CVP. If CVP is assumed to be 5 mm Hg, this gives a sPAP of 37 mm Hg which amounts to a mPAP of 24 mm Hg based on the prediction equation 0.6 × sPAP + 2 = mPAP [113].

^§^Calculated mPAP based on the prediction equation 0.6 × sPAP + 2 = mPAP [113].
